# Follow-up after bariatric surgery: is it time to tailor it? Analysis of early predictive factors of 3-year weight loss predictors of unsuccess in bariatric patients

**DOI:** 10.1007/s13304-022-01314-5

**Published:** 2022-07-02

**Authors:** Costantino Voglino, Simona Badalucco, Andrea Tirone, Cristina Ciuoli, Silvia Cantara, Nicoletta Benenati, Annalisa Bufano, Caterina Formichi, Federica Croce, Ilaria Gaggelli, Maria Laura Vuolo, Giuseppe Vuolo

**Affiliations:** 1 Department of General and Specialized Surgery, Unit of Bariatric Surgery, University of Siena, Policlinico “Le Scotte”, Viale Bracci 14, 53100 Siena, Italy; 2 Department of Medicine, Surgery and Neurosciences, Unit of Endocrinology, University of Siena, Viale Bracci, Policlinico “Le Scotte”, 53100 Siena, Italy; 3 Department of Medicine, Surgery and Neurosciences, University of Siena, Viale Bracci, Policlinico “Le Scotte”, 53100 Siena, Italy; 4 Department of Diagnostic Imaging, Hospital Campostaggia, Campostaggia, 53036 Poggibonsi, SI Italy

**Keywords:** Bariatric surgery, Weight loss, Sleeve gastrectomy, Predictors, Follow-up

## Abstract

Bariatric surgery (BS) is the most effective treatment strategy for obesity. Nevertheless, a subset of patients does not reach a successful weight loss or experience long-term weight regain. Conflicting evidence exists regarding predictors of BS outcomes. We aimed to define the early factors linked to 3 year unsuccessful weight loss in order to promote a tailored close follow-up. We enrolled 443 patients who underwent BS from January 2014 to December 2018 with a 3 year follow-up. An unsuccessful BS outcome was defined as a percentage of total weight loss (%TWL) < 20. We compared the characteristics between successful and unsuccessful patients in order to identify predictor factors of unsuccess after surgery. We found that the proportion of patients with unsuccessful weight loss progressively increased from one to three years after BS. In a multiple regression model, only 1 month %TWL and sleeve gastrectomy (SG) were significantly associated with 3 year unsuccessful weight loss. We stratified our cohort in four groups according to the risk of BS unsuccess, in terms of 1 month %TWL and type of surgery (SG vs gastric bypass). Interestingly, groups showed a significant difference in terms of %TWL at each follow-up point. Patients submitted to SG with lower 1 month %TWL must be considered at higher risk of future weight regain; consequently, they require a tailored and closer follow-up.

**Graphical abstract:**

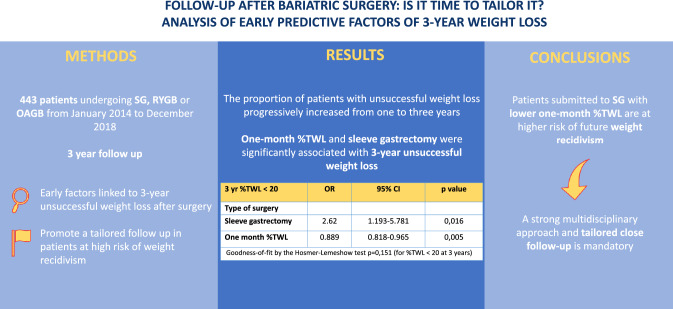

## Introduction

The worldwide obesity rate continues to grow and it is a significant issue for individuals and the healthcare system. According to the WHO data, obesity affects about 13% of the world’s adult population and its prevalence has nearly tripled since 1975 [1]. Bariatric surgery (BS) is the most effective treatment strategy that results in significant and sustained long-term weight loss and amelioration/remission of obesity-related comorbidities [2–4]. Despite marked weight loss following BS, there is a subset of patients that fails to achieve a successful weight loss or experiences long-term weight regain. In fact, in our 3 year follow-up previous paper we reported the highest value of %Excess Weight Loss (%EWL) at 12 months after surgery and, after this time, a slight but constant decrease of %EWL with the lowest value observed at 3 years [5].

The definition of BS success/unsuccess is variably reported in literature. % EWL is one of the commonly used measures of BS outcome. In particular, the EWL > 50% was extensively reported as a criterion for BS success.

However, we have decided to use % Total Weight Loss (%TWL) as anthropometric outcome measure of choice because it was, in contrast to % EWL, independent or anyway less influenced by pre-operative BMI. According to literature BS success was defined as TWL > 20% [6].

Social and demographic features, surgical procedures, mental and eating disorders, and pre-operative anthropometric variables are usually reported as weight loss predictor factors [7–9]. Despite numerous studies in this area, few robust predictors of BS unsuccess have been clearly established. Defining the early factors linked to BS unsuccess is mandatory to promote prompt intervention in order to optimize weight loss.

The primary aim of this study was to identify clinical, demographic, and/or anthropometric variables associated with poor %TWL during a 3 year follow-up period.

## Materials and methods

### Study population

We performed a retrospective analysis of 443 patients who underwent BS for morbid obesity in the Unit of Bariatric Surgery of Siena from January 2014 to December 2018. Inclusion criteria for BS are as follows:

1 body mass index (BMI) > 40 kg/m2 or > 35 kg/m2 with co-morbidities, 2 patients ranging from 18 to 65 years old. We discussed all of the cases in a multidisciplinary team which includes a surgeon, an endocrinologist, a bariatric dietitian, and a psychiatrist specialized in obesity and eating disorders in order to decide whether to perform BS and the type of surgical procedure. Patients with incomplete follow-up or missing data were excluded from the present study.

This research was approved by Ethics Committee of our Hospital. Informed consent was obtained from all patients.

We recorded academic and socio-demographic factors and anthropometric data of each patient. Their medical history and previous obesity-related comorbidities, included eating and mental disorders, were also evaluated.

### Surgical technique

All surgical procedures were performed with a minimally invasive approach by trained surgeons with advanced and comparable skills in bariatric surgery in order to avoid some relevant bias related to the experience of the operating team.

### Sleeve gastrectomy (SG)

The procedure started with a dissection of the gastrocolic ligament. The greater curve was skeletonized from 5 cm to pylorus up to the angle of His. A complete mobilization of the gastric fundus was achieved. The left lateral portion of the stomach was resected using a 60-mm linear stapler along a bougie (36–38 Fr). At the end of the procedure, resected stomach was removed through left trocar site.

### One anastomosis gastric bypass (OAGB)

Using a 60 mm linear stapler, a long and narrow gastric pouch was created. A 38-Fr bougie was used as a guide for calibration. Finally, an antecolic Billroth II-type loop gastroenterostomy was done at the small bowel 200 cm distal to the Treitz ligament.

### Roux-en-Y gastric bypass (RYGB)

We began the operation by creating a stomach pouch of approximately 30 ml along a 38-Fr calibration bougie. The Roux reconstruction was performed with double loop technique. The biliopancreatic limb was set to 80 cm distal to the Treitz ligament, while the alimentary limb length was set to 150 cm. For gastrojejunostomy we carried out a side-to-side anastomosis using a mechanical stapler. A side-to-side jejunojejunostomy was performed between alimentary and biliary limbs. Finally, the biliary loop and alimentary loop were separated using a linear stapler.

### Follow-up

After discharge, a personalized diet and post-operative follow-up schedule have been provided to all patients. Patients were followed up in our outpatients department by a multidisciplinary team. Medical visits were scheduled at 1, 3, 6, and 12 months in the first year, then every 6 months for the following year, and then every 12 months.

### Statistical analysis

Variables not normally distributed are expressed as median and interquartile range (IQR, i.e., the range between 25 and 75th percentile) and were compared by means of non-parametric test (Mann–Whitney). The *χ*2 test or Fisher exact test was used to compare categorical variables. A multinomial logistic regression model was performed in order to identify the variables associated with unsuccessful BS.

The Hosmer–Lemeshow test was used to evaluate the goodness-of-fit of the multivariate models.

Statistical significance was determined at *P* value of < 0.05. For statistical analysis, SPSS statistical package (version 20.0) (SPSS™, Chicago, Illinois, USA) was used.

## Results

Four hundred forty-three patients were included in our study, of which 342 (77.2%) were female. Two hundred forty-one patients (54.4%), 123 (27.7%), and 79 (17.8%) underwent sleeve gastrectomy, RYGB, and OAGB, respectively. Baseline patients’ characteristics are summarized in (Table 1). The median age of patients was 44 years old and the median pre-operative BMI was 45.5 (IQR 41.6–51). A history of eating disorders and mental disorders was present in 50.8 and 31.8%, respectively. A surprising 41.3% of our cohort was unemployed, homemakers, or retired. The majority of patients (90.3%) had almost one obesity-related comorbidity. Steatosis had been diagnosed in 61.2% of patients. Hypertension was reported in 42.4% of our sample. The 3 year remission rate of Type 2 Diabetes was reported in 86.3, 88, and 89.3% in SG, OAGB, and RYGB, respectively (*p* > 0.05). Sleeve gastrectomy achieved a lower dyslipidemia remission rate than OAGB and RYGB at 3 year (46.4, 70.6, and 74.4% in SG, OAGB, and RYGB group, respectively; *p* < 0.05). After 3 years, 47.7% of SG, 50% of OAGB, and 50.8% of RYGB had experienced arterial hypertension remission (*p* > 0.05).

**Table 1:** Baseline characteristics of 443 patients

Parameter	Total
Patients	443
Sex (male; female)	101; 342 (22.8%; 77.2%)
Type of surgery	
Sleeve gastrectomy	241 (54.4%)
OAGB	79 (17.8%)
RYGB	123 (27.7%)
Age (median, IQR)	44 (36;52)
Place of birth	
South Italy	111 (25.1%)
Central Italy	260 (58.7%)
North Italy	14 (3.2%)
Insular Italy	33 (7.4%)
Foreign country	25 (5.6%)
Place of current residence	
South Italy	39 (8.8%)
Central Italy	396 (89.4%)
North Italy	8 (1.8%)
Marital status	
Married or living with a partner	289 (65.2%)
Divorced, widowed or single	154 (34.8%)
Job	
Employee	260 (58.7%)
Unemployed, housewives, retired	183 (41.3%)
Educational qualifications	
Highschool or higher	200 (45.1%)
Secondary school or lower	243 (54.9%)
Dyslipidemia (yes; no)	224; 219 (50.6%; 49.4%)
Arterial Hypertension (yes; no)	188; 255 (42.4%; 57.6%)
Steatosis (yes; no)	271; 172 (61.2%; 38.8%)
Sleep apnea (yes; no)	128; 315 (28.9%; 71.1%)
Type 2 diabetes (yes; no)	141; 302 (31.8%; 68.2%)
Total comorbidities (0–5)	
0	43 (9.7%)
1	101 (22.8%)
2	132 (29.8%)
3	95 (21.4%)
4	58 (13.1%)
5	14 (3.16%)
Mental disorders (yes; no)	141; 302 (31.8%; 68.2%)
Eating disorders (yes; no)	225; 218 (50.8%; 49.2%)
Pre-operative BMI (median; IQR)	45.5 (41.6–51)
One month %TWL	11.1 (9–13.2)

**Fig. 1:**
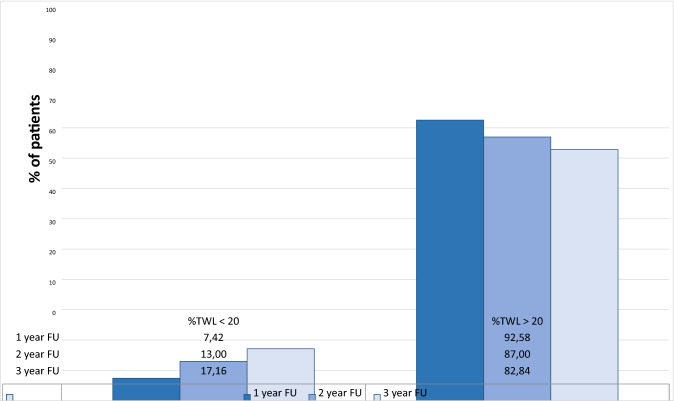
Proportion of unsuccessful bariatric patients from one to three-year follow-up course

**Fig. 2:**
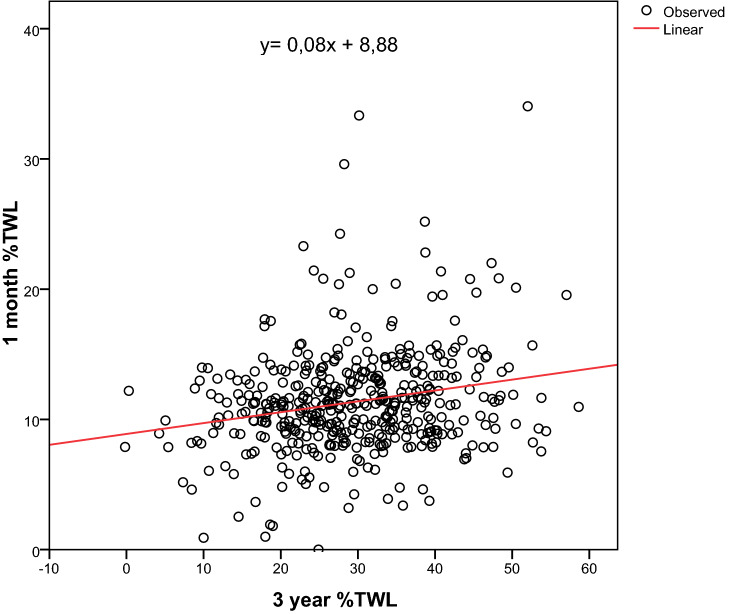
Linear correlation between one-month %TWL and three-year %TWL (*p* < 0.01)

As expected, the proportion of patients with unsuccessful weight loss progressively increased from one to three years, being 7.42, 13, and 17.16% at 12, 24, and 36 months after BS, respectively (Fig. 1). According to the 3 year %TWL, patients were divided into two groups: unsuccessful patients (%TWL < 20) and successful patients (%TWL > 20) (Table 2). Older age, rate of steatosis and sleep apnea, higher pre-operative BMI, type of surgery, place of birth, and number of total comorbidities were associated with unsuccessful BS. We also found a linear significant correlation between 1 month %TWL and 3 year %TWL (*p* < 0.01) (Fig. 2). The linear regression was estimated by the formula: *y* = 0.08x + 8, 88.

**Table 2:** Analysis of social, demographic and clinical parameters in unsuccessful patients (%TWL < 20), and successful patients (%TWL > 20) at 3 year follow-up

Parameter	3 year %TWL < 20	3 year %TWL > 20	*p* value
Patients	76 (17.2%)	367 (82.8%)	
Sex (male; female)	17; 59 (16.8%; 17.3%)	84; 283 (83.2%; 82.7%)	0.528
Type of surgery			< 0.01
Sleeve gastrectomy	62 (25.7%)	179 (74.3%)	
OAGB	4 (5.1%)	75 (94.9%)	
RYGB	10 (8.1%)	113 (91.9%)	
Age (median, IQR)	49 (43–55)	43 (36–50)	< 0.001
Place of birth			0.018
South Italy	13 (11.7%)	98 (88.3%)	
Central Italy	45 (17.3%)	215 (82.7%)	
North Italy	2 (14.3%)	12 (85.7%)	
Insular Italy	10 (30.3%)	23 (69.7%)	
Foreign country	6 (24%)	19 (76%)	
Place of current residence			0.091
South Italy	4 (10.3%)	35 (89.7%)	
Central Italy	72 (18.2%)	324 (81.8%)	
North Italy	0	8 (2.2%)	
Marital status			0.308
Married or living with a partner	52 (18%)	130 (84.4%)	
Divorced, widowed or single	24 (15.6%)	237 (82%)	
Job			0.230
Employee	48 (18.5%)	212 (81.5%)	
Unemployed, housewives, retired	28 (15.3%)	155 (84.7%)	
Educational qualifications			0,381
Highschool or higher	36 (18%)	164 (82%)	
Secondary school or lower	40 (16.5%)	203 (83.5%)	
Dyslipidemia (yes;no)	41; 35 (18.3%; 16%)	183; 184 (81.7%; 84%)	0.301
Arterial Hypertension (yes;no)	35; 41 (18.6%; 16.1%)	153; 214 (81.4%; 83.9%)	0.282
Steatosis (yes;no)	59; 17 (21.8%; 9.9%)	212; 155 (78.2%; 90.1%)	0.001
Sleep apnea (yes;no)	31; 45 (24.2%; 14.3%)	97; 270 (75.8%; 85.7%)	0.01
Type 2 diabetes (yes;no)	30; 46 (21.3%; 15.2%)	111; 256 (78.7%; 84.8%)	0.077
Total comorbidities (0–5)			0.001
0	4 (9.3%)	39 (90.7%)	
1	11 (10.9%)	90 (89.1%)	
2	24 (18.2%)	108 (81.8%)	
3	16 (16.8%)	79 (83.2%)	
4	16 (27.6%)	42 (72.4%)	
5	5 (35.7%)	9 (64.3%)	
Mental disorders (yes;no)	29; 47 (20.65%; 15.6%)	112; 255 (79.4%; 84.4%)	0.122
Eating disorders (yes;no)	39; 37 (17.3%; 17%)	186; 181 (82.7%; 83%)	0.510
Pre-operative BMI (median;IQR)	44.2 (40.4–48.1)	46.1 (41.9–51.7)	0.011
One month %TWL (median;IQR)	10.4 (8.2–11.9)	11.2 (9.2–13.5)	0.013

**Fig. 3:**
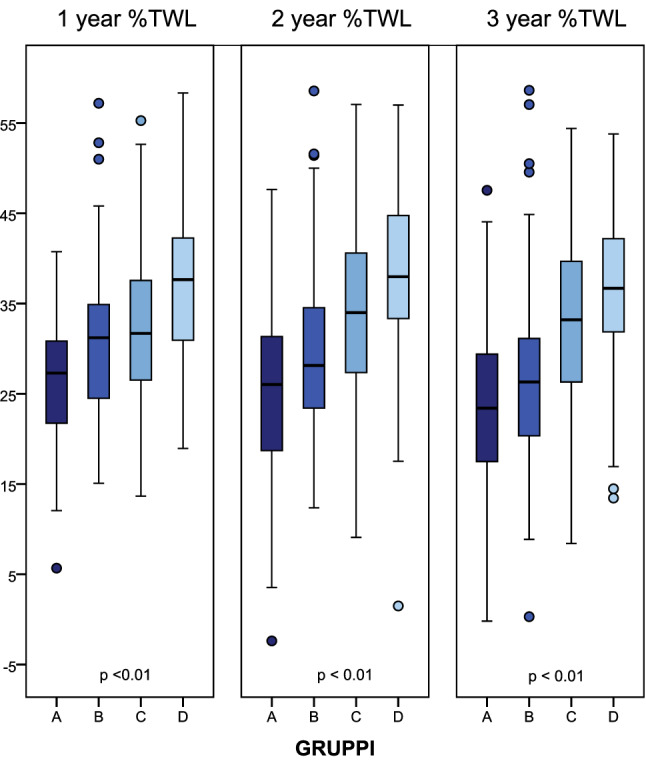
%TWL at different follow-up point among four groups (group **A**: patients underwent SG with 1 month %TWL < 10.48; group *B*: patients underwent SG with 1 month %TWL > 10.48; group **C**: patients underwent RYGB/OAGB with one-month %TWL < 10.48; group **D**: patients underwent RYGB/OAGB with 1 month %TWL > 10.48)

We performed a multivariate analysis, including the variables that resulted significant from the bivariate analysis. In a multiple regression model only sleeve gastrectomy and lower %TWL at 1  month remained significantly associated with 3 year unsuccessful weight loss (Table 3). On the basis of these parameters, we stratified patients according to the risk of BS unsuccess. For calculating the cut-off of 1 month %TWL that predicts 3 year BS success, we assigned the value of 20 to the variable *x* in the above-mentioned formula. We obtained *y* = 10.48. We divided our cohort in four groups: group A (patients underwent SG with 1 month %TWL < 10.48); group B (patients underwent SG with one-month %TWL > 10.48); group C (patients underwent RYGB/OAGB with 1 month %TWL < 10.48); and group D (patients underwent RYGB/OAGB with 1 month %TWL > 10.48). Interestingly, groups showed significant difference in terms of %TWL at each follow-up point (Fig. 3).

**Table 3:** Association between sleeve gastrectomy and lower one month %TWL and 3 year unsuccessful weight loss in a multiple regression model

3 year %TWL < 20	OR	95 % CI	*p* value
Type of surgery			
Sleeve gastrectomy	2.62	1.193–5.781	0.016
One month %TWL	0.889	0.818–0.965	0.005

Goodness-of-fit by the Hosmer–Lemeshow test *p* = 0,151 (for %TWL < 20 at 3 years)

## Discussion

To date, BS is widely considered the most reliable therapeutic strategy which results in significant and sustainable weight loss, improvement of obesity-related comorbidities, and prolonged survival. However, a subset of patients after BS experiences unsuccessful weight loss or weight regain. This issue has important health consequences for both the patients (decreased quality of life and working force) and the public health system in terms of costs associated with managing remerging obesity and the related comorbidities. Early identification of patients who will undergo BS unsuccess may allow intensive post-operative behavior and lifestyle interventions and strict follow-up in order to maximize weight loss. In our research, we have found that the type of surgery and 1 month %TWL were associated with BS failure. These results suggested that patients at risk of BS unsuccess could be identified in the pre-operative as well as in the early post-operative period.

Several authors have investigated the relationship between pre-operative BMI and post-operative weight loss after BS. Multiple studies showed that higher pre-operative BMI was correlated with worse weight trajectory. In a recent paper by Nickel and colleagues [10], the predictors of %EWL 12 months after BS were analyzed. They reported a negative significant correlation between pre-operative BMI and weight loss after surgery. Others show similar results [11–13]. In our report, we did not identify any association between pre-operative BMI and weight loss. This result could be justified by the different anthropometric outcome measures of choice that we decided to use. In fact, we preferred %TWL, instead of %EWL, because it is less influenced by pre-operative BMI. On the contrary, %EWL is strongly and negatively associated with baseline BMI [14, 15].

Reports in literature on difference in weight loss between males and females after BS are spare and conflicting. In line with D’Eusebio et al. and Obanda and colleagues we did not identify gender as a pre-operative predictor of outcome [16, 17]. Conversely, several authors reported male gender as a predictive factor of less favorable weight trajectory [18, 19]. The correlation between age and BS unsuccess is still a matter of debate. Multiple authors found an inverse significant association between age and weight loss [11, 20–22]. In a large nationwide study involving over 2000 patients who attended 5 year follow-up, Dreber and colleagues showed a greater weight loss in younger patients [23]. However, we failed to find any correlation between age and %TWL at 36 months after surgery in line with other authors [17, 24]. It is well known that increasing age is strongly linked to more comorbidities, limitation of physical activity, reduction of compliance to diet and lifestyle recommendations, and lower resting energy expenditure. Therefore, these variables could act as confounding factors leading to heterogeneous results in literature. In addition, we would emphasize that the goals of BS should be different according to the age. In older patients, who have lower life expectancies than younger patients, the primary aim of surgery is to mitigate the effect of comorbidities in order to provide important health benefits regardless of weight loss. Younger obese patients suffer from social stigma and discrimination, which has a negative impact on mental health, socialization, and attitude toward school and employment status outcomes [25]. Therefore, in these people the purpose is not only to achieve a significant improvement of obesity-related disease for many decades but also to reach a substantial and sustainable weight loss.

The relationship between the above-mentioned mental health issue, employment status and the educational qualification, and the weight loss after BS has been extensively investigated by several authors, but the results are controversial.

Dawes and colleagues, in a recent meta-analysis, have investigated the correlation between pre-operative mental health disorders and post-operative weight loss [26]. In consistent with our results, the authors did not find clear evidence that pre-operative psychopathologic condition was related to BS unsuccess.

Emotional eating and other problematic eating behaviors are common among BS patients, but it is unclear how they may affect post-surgical outcomes. We failed to find any correlation between eating disorders and BS success in line with some studies [27, 28] but conversely to others [29, 30]. A published study showed that post-operative eating factors could play an important role in determining post-surgical weight loss more than pre-operative eating behavior [31]. In fact, post-operative grazing and uncontrolled eating are consistently associated with insufficient weight loss or weight regain [32]. In the light of the above, we would stress the importance of appropriate and close nutritional and psychiatric follow-up with the aim of preventing weight regain and/or insufficient weight loss and achieving a long-term body weight stability.

In Italy, a North–South gradient is present with a higher prevalence of obesity in southern and insular regions in contrast with the north and center. The factors taken into account to explain this geographical trend include regional eating habits and socio-economic status [33–35]. Moreover, many studies reported the association between overweight and obese patients and educational attainment, but it still remains unclear whether a lower educational level is a contributing cause or effect of obesity [36]. On this basis, we decided to investigate the relationship between BS unsuccess and geographical origin, place of current residence, and educational qualification. In line with a recent paper, in our study a lower level of education was not a predictor of worse outcome [17]. Also geographical factors were not associated with weight loss after surgery.

We found an increased number of comorbidities among patients with lower 3 year %TWL. However, the association not remained significant in a multivariate model. Our results are similar with previous studies [17, 20].

The magnitude of BS unsuccess after different bariatric procedures was variably reported in literature. A recent meta-analysis by Hu et al. reported that patients underwent SG experienced a worse outcome with regard to 3 year %EWL [37].

Furthermore, Chang and colleagues in a retrospective 5 year follow-up study of 247 patients who underwent SG or RYGB showed that SG is an independent risk factor of insufficient weight loss [38]. In our previous paper, we reported the non-inferiority of OAGB, in terms of 3 year %TWL, compared to RYGB [5].

In our series, SG was predictor of BS unsuccess in a multivariate analysis. This highlights the importance of clearly informing expectations and planning strict and close interventions.

As already suggested by our previous work, early weight loss is one of the stronger predictors of outcomes in terms of weight loss [39]. The explanation for this phenomenon remains unclear and not completely understood. In our cohort all patients were discharged with the same diet program and post-operative follow-up schedule. According to our experience, in the first month after surgery, the adherence to the post-operative recommendations is high, and therefore, the impact of confounding factors is minimal. The reasons for the different early outcomes should be researched in the hormonal changes caused by surgery and genetic factors. Similar findings were shown by Obeidat et al. in a cohort of 190 patients who underwent SL [40]. A strong correlation between %EWL at 6 months and %EWL at 12 and 24 months was demonstrated by D’Eusebio and colleagues [16].

An early identification of patients who will fall below the normal curve for weight loss in the pre-operative/early post-operative period should be mandatory. Based on these premises, a promising strategy will be to tailor follow-up in order to improve outcomes.

In fact, an ideal and adequate follow-up program should be individualized according to the predicted risk and timing of insufficient weight loss/weight regain.

For this purpose, we proposed a stratification of bariatric patients in four groups of risk depending on the type of surgery and 1 month %TWL. An intensive follow-up program should be reserved for patients at high risk of weight regain with the aim of promoting a prompt multidisciplinary approach, including nutritional and psychiatric counseling, behavior intervention, and pharmacotherapy to maximize weight loss and minimize the costs of health system [41].

So far, we identify only one previous paper focused on BS unsuccess after three types of surgery [17]. A strength of our study was the size of the cohort with a complete 3 year follow-up. A further strength consists in our use of %TWL as a metric of choice for assessing weight loss across the bariatric population because it is the least influenced by pre-operative BMI. Furthermore, we first proposed a tailored follow-up program in patients at high risk of unsuccessful results after BS.

Nevertheless, our study has some limitations: it is a retrospective study with a relative short 3 year follow-up; we have not analyzed some factors as change of feed, physical activity, and diet adherence, which may affect weight loss.

## Conclusion

In conclusion patients submitted to SG with lower 1 month %TWL must be considered at higher risk of future weight regain; therefore, they require a strong multidisciplinary approach and tailored close follow-up with the aim of improving their outcomes. Longer follow-up studies would be desirable to collect stronger data.

Further studies are needed to better identify the predictor factors of BS unsuccess focusing on hormonal changes and epigenetic features that could explain the huge differences regarding weight loss recorded since the first post-operative month and maintained up to three years after surgery.
